# Profile of People Living with HIV Switching Prior Antiretroviral Treatment to a Doravirine-Based Regimen in the Real-World Clinical Setting in Greece: The Retrospective DORAVITO Study

**DOI:** 10.3390/biomedicines14081761

**Published:** 2026-08-05

**Authors:** Antonios Papadopoulos, Myrto Astriti, Vasileios Papastamopoulos, Vassileios Paparizos, Helen Sambatakou, Symeon Metallidis, Konstantinos Protopapas, Charalampos Moschopoulos, Georgios Adamis, Panagiota Lourida, Charisis Totsikas, Varvara Vasalou, Theofilos Chrysanthidis, Panagiotis Kollaras, Eleni Boutselakou, Dimitris Tsokos, Georgios Trimis, Lazaros Poughias

**Affiliations:** 1 4th Department of Internal Medicine, University General Hospital Attikon, National and Kapodistrian University of Athens, 124 61 Athens, Greece; antpapa1@otenet.gr (A.P.); kprotopapas@hotmail.com (K.P.); bmosxop@yahoo.gr (C.M.); 2 1st Department of Internal Medicine and Infectious Diseases, G. Gennimatas General Hospital of Athens, 115 27 Athens, Greece; myrto_astriti@hotmail.com (M.A.); geo.adamis@gmail.com (G.A.); 3 5th Department of Internal Medicine, Division of Infectious Diseases, Evangelismos General Hospital of Athens, 106 76 Athens, Greece; vasileios.papastamopoulos@gmail.com (V.P.); giotalourida@gmail.com (P.L.); charisistotsikas@gmail.com (C.T.); 4 HIV Unit, 1st Department of Dermatology and Venereology, Medical School, National and Kapodistrian University of Athens, “A. Sygros” Hospital for Skin and Venereal Diseases, 161 21 Athens, Greece; vpaparizos@yahoo.gr (V.P.); vvasalou@gmail.com (V.V.); 5 HIV Unit, Second Department of Internal Medicine, Hippokration General Hospital of Athens, 115 27 Athens, Greece; esabatak@med.uoa.gr; 6 Aristotle University HIV Unit, AHEPA University Hospital, 546 36 Thessaloniki, Greece; metallidissimeon@yahoo.gr (S.M.); teochriss@yahoo.co.uk (T.C.); p.kollaras@gmail.com (P.K.); 7 MSD Greece, Global Medical & Scientific Affairs, 174 56 Athens, Greece; eleni.boutselakou@msd.com (E.B.); georgios.trimis@msd.com (G.T.); lazaros.poughias@msd.com (L.P.)

**Keywords:** antiretroviral, doravirine, HIV, real-world, simplification, switch

## Abstract

**Background:** Doravirine (DOR) is a new-generation non-nucleoside reverse transcriptase inhibitor, which has emerged as an option for people living with HIV (PLWH) owing to its favorable efficacy and safety profile, unique resistance pathway and limited potential for drug-drug interactions. **Methods:** This retrospective chart review study aimed to better understand DOR-based treatment use in Greece, PLWH characteristics, and drivers of treatment switch. Eligible individuals were adult PLWH who were switched to a DOR-based regimen based on the physician’s decision. Individuals exposed to DOR at any time prior to switching to the DOR-based regimen were excluded. **Results:** From 12 July 2023 to 31 October 2023, 110 PLWH were consecutively enrolled across 6 public hospital clinics. At baseline (closest prior to or on the date of first DOR prescription), the mean age of PLWH was 49.3 years, 90.9% were males, 88.2% were asymptomatic, 86.5% were virologically suppressed, 33.6% were suffering from multimorbidity (excluding infections/infestations), 45.5% were receiving comedications for their comorbidities, and 5.5% were co-infected with Hepatitis C virus. Most PLWH (98.2%) were prescribed DOR plus two nucleoside reverse transcriptase inhibitors; 87.3% were prescribed DOR/Lamivudine/Tenofovir Disoproxil Fumarate fixed-dose combination. PLWH started DOR a median of 11.7 years after first-ever antiretroviral therapy initiation, corresponding to 2nd/3rd/≥4th antiretroviral line in 33.6%/33.6%/32.7% of participants, respectively; 60.9% of them were proactively switched to a DOR-based regimen. The most common reasons for switching were ‘regimen simplification’ (42.7%), ‘tolerability’ (26.4%) and ‘prevention of toxicities’ (18.2%). **Conclusions:** This study highlights the patterns of DOR use in real-life clinical practice in Greece among treatment-experienced PLWH. Physicians switch HIV-1-infected individuals from prior ART to DOR-based regimens to offer a simplified regimen or to avoid or prevent toxicity.

## 1. Introduction

Infection with human immunodeficiency virus (HIV) can progress to acquired immunodeficiency syndrome (AIDS) with an inevitable fatal outcome if left untreated [1,2,3]. In the absence of curative options, current HIV management requires lifelong treatment administration, with rising concerns of inadequate treatment adherence and an increased risk of developing non-AIDS comorbidities, such as metabolic disorders and cardiovascular (CV) diseases [3,4,5,6,7]. These concerns, taken together with the high prevalence of 39.9 million people living with HIV (PLWH) globally in 2023 [8], suggest that HIV will continue to be a major threat to public health. By the end of 2025, an estimated total of 21,918 HIV cases had been diagnosed in Greece since the start of the AIDS epidemic [9].

A large number of antiretrovirals (ARVs) are currently available [10]. Current European antiretroviral treatment (ART) guidelines recommend 2 nucleos(t)ide reverse transcriptase inhibitors (NRTIs) as a backbone plus a nucleoside reverse transcriptase inhibitor (NNRTI) or an integrase strand transfer inhibitor (INSTI)-based regimen as first-line, which are proven to be effective at virological suppression [10]. A regimen can be modified in case of intolerance, toxicity, aging and/or comorbidities, drug-drug interactions (DDIs), simplification issues, dietary needs or limitations, and cost reduction [10].

DOR is an NNRTI administered orally as a once-daily (QD) treatment for HIV-1 infection [11,12]. Its features include the lack of food restriction and significant DDIs (e.g., with proton-pump inhibitors), a better neuropsychiatric profile compared to efavirenz (EFV), a better lipid profile than RTV-boosted darunavir (DRV) and EFV, and the lack of impact on the corrected QT interval [13,14]. It has demonstrated antiviral activity in vitro against the most common NNRTI resistance-associated mutations (RAMs). It has a distinct resistance profile compared with first-generation NNRTIs [15,16,17,18,19], with a low prevalence of DOR-specific RAMs (0.7–1.8% in treatment-naive and 6.1–18.2% in treatment-experienced PLWH) based on European real-world studies (including data from France, Greece, Italy, Poland and Spain) [20,21,22,23,24,25].

In Europe, DOR is available both as a single-agent tablet marketed as Pifeltro^®^ and as a single-tablet regimen (STR) with lamivudine (3TC) and tenofovir disoproxil fumarate (TDF), marketed as Delstrigo^®^ [11,12]. DOR is indicated for the treatment of infection with HIV without past or present evidence of resistance to the NNRTI class; it is recommended for both treatment-naive and virologically suppressed PLWH [10,26,27,28,29]. The randomized, phase 3 DRIVE-SHIFT trial demonstrated that DOR/3TC/TDF is an effective, non-inferior, generally well-tolerated option for maintaining viral suppression in PLWH considering a change in ART [28,29]. The clinical trial findings among PLWH switching to DOR-containing regimens have since been supported by several real-world studies across Europe [25,30,31,32,33,34,35,36,37,38,39,40], including a favorable metabolic impact [33,35], a promising reduction in 10-year risk of CV disease [33], and a neutral impact on weight gain [35].

DOR is a potent and effective NNRTI that is emerging as an increasingly attractive option for PLWH, including those considering a change in therapy. However, at the time of study design, informative real-world evidence (RWE) on reasons for switching to DOR-containing regimens in Europe was scarce (Italy [25,35,41,42], England [36], Spain [37], and The Netherlands [38]), while Greek data on DOR use was lacking. Therefore, the DORAVITO multicenter, retrospective study aimed to characterize the profile of PLWH considered suitable for switching to a DOR-based regimen, and to understand the rationale underpinning ARV switch to a DOR-based regimen in routine care settings in Greece.

## 2. Methods

### 2.1. Study Design and Population

DORAVITO was a multicenter, retrospective, observational switch cohort study with a baseline snapshot based on secondary data collection via medical chart review. The aim was to obtain a representative sample of PLWH treated with DOR-based regimens in real-world settings of Greece. It was carried out by hospital-based infectious disease specialists who treat PLWH in major national HIV centers.

Eligible individuals were adult PLWH at the time of informed consent, who were switched to a DOR-based regimen based on the physician’s decision, and who had sufficient available medical records for data abstraction to meet the primary objectives of the study. The switch to a DOR-based regimen should have occurred in the post-marketing setting and ≥30 days before site initiation. Deceased individuals and individuals exposed to DOR at any time in the past before switching to the DOR-based regimen were excluded from the study.

Any deviation from the approved labeling of the treatment components of the DOR-based regimen was not considered a reason for exclusion of a PLWH from the study provided that the physician determined it was in the best interest of the PLWH; it did not pose any undue safety risk to the PLWH; and it was reported to the sponsor as off-label use.

To minimize sampling bias, physicians were requested to consecutively, thus non-selectively, include the first eligible individuals that met the study-specific eligibility criteria, either identified through medical chart review or attending their clinic during a routine visit. Physicians were also asked to provide documentation of the screening process results for both consented and non-consented individuals in order to assess potential bias after recruitment termination.

The retrospective observation period for each participant extended to the date of initial HIV-1 diagnosis and ended on the date of first prescription of the DOR-based regimen; no data after that date were collected in the context of this study. Baseline represented the timepoint closest prior to or on the date of first prescription of the DOR-based regimen.

The study was designed, conducted, and reported in alignment with the ethical principles of the Declaration of Helsinki, the Good Pharmacoepidemiology Practices (GPP) guidelines of the International Society for Pharmacoepidemiology (ISP) [43], the STROBE (Strengthening the Reporting of Observational Studies in Epidemiology) guidelines where applicable [44], the European Union General Data Protection Regulation [45], as well as relevant local rules and regulations.

### 2.2. Study Objectives

The primary objectives of this study were to describe: (1) the baseline sociodemographic, anthropometric, lifestyle, disease and ART characteristics, as well as the comorbidity and comedication burden, among HIV-1-infected individuals switched from a prior ART to a DOR-based regimen, and (2) the factors that guide clinicians’ decision to proactively or reactively switch PLWH from a prior ART to a DOR-based regimen, in routine clinical practice in Greece.

Proactive switch was defined as ‘switching to the new regimen in the absence of therapeutic failure, documented intolerance and patient’s non-adherence.’ Reactive switch was defined as ‘switching to the new regimen due to documented therapeutic failure, intolerance/toxicity and/or patient’s non-adherence.’ The reasons for switching were structured as a predetermined multiple-choice list of options, all of which are listed verbatim in the Results.

Secondary objectives were to present the aforementioned information per prescribed DOR formulation, per combined drug class with DOR, and per ART line setting. The subgroups by drug class included PLWH switched to DOR in combination with 2 NRTIs and those switched to DOR with ARVs other than NRTIs. This subgrouping reflects distinct switching strategies tailored to individual PLWH characteristics, including treatment history, resistance profiles, and clinical needs.

As an exploratory objective, the study aimed to capture the prevalence of HIV-1 RAMs in the subgroup of PLWH that had undergone genotypic resistance testing (GRT) at baseline.

### 2.3. Sample Size Determination

The study was purely descriptive and was not planned to reject or affirm any formal statistical hypothesis. Taking into consideration the recent introduction of DOR in the Greek market (4 February 2022) at the time of study design, and based on feasibility projections (i.e., site’s recruitment potential and number of potentially eligible patients), a sample size of 100 eligible patients was considered both feasible and sufficient for estimating the primary outcome measures. A sample of 100 offers a maximum margin of error of 10.2% [with 95% confidence intervals (CIs) using the Clopper-Pearson exact method ranging between 39.8% and 60.2%] for the estimation of a percentage of 50%, where the width of the CI is the largest, therefore ensuring an acceptable level of precision to draw meaningful conclusions. This means that for any qualitative variable observed at any frequency (0% to 100%), the precision will range from 3.6% to 10.2% at a 95% CI.

The study actually included 110 eligible PLWH, thus ensuring an even smaller margin of error. Nevertheless, the size of the planned subgroups was smaller, limiting any inferential interpretation with regard to the secondary outcome measures. Thus, any descriptions or comparisons between subgroups are purely narrative.

### 2.4. Data Collection

Data were extracted from medical records and documented in standardized electronic Case Report Forms (eCRFs) incorporated into a project-specific web-based data capture (WBDC) platform. The WBDC platform adhered to all applicable data protection regulations and requirements with regard to electronic records and database validation.

Data were entered electronically onsite, via the WBDC platform using a web-based secure server, by trained, authorized users (the physician/investigator or a qualified designee) on a real-time basis with the use of a standard web browser. Access to the data was restricted and monitored through password protection and an audit trail.

Quality control mechanisms and processes were implemented to ensure that all clinical data entered in the database was valid, complete and accurate for statistical analysis. A query management process was in place for handling the eCRF-collected data on a real-time-basis through built-in edit and logic checks (validation rules) in the eCRF application. In addition, comprehensive data cleaning activities were performed prior to the final analysis. For any discrepancies (other than those predicted by the built-in validation rules) that arose during the source data verification process or data review, queries were handled electronically through the specific query functionality of the eCRF application.

### 2.5. Statistical Analyses

Statistical Analyses were only of descriptive nature; categorical variables are presented as absolute and relative frequencies, whereas continuous variables are summarized as mean and standard deviation (SD) for normally distributed data or median and interquartile range (IQR) for non-normally distributed data, as examined using the Shapiro-Wilk test. The exact Clopper-Pearson CIs were calculated for binomial proportions where specified. All statistical tests were two-sided and performed at a 0.05 significance level.

No imputation methods were applied with the exception of partial dates. The sample size determination and statistical analyses were performed using SAS^®^ software, Version 9.4 (SAS Institute Inc., Cary, NC, USA).

## 3. Results

### 3.1. Disposition and Treatment Characteristics

**Figure 1:**
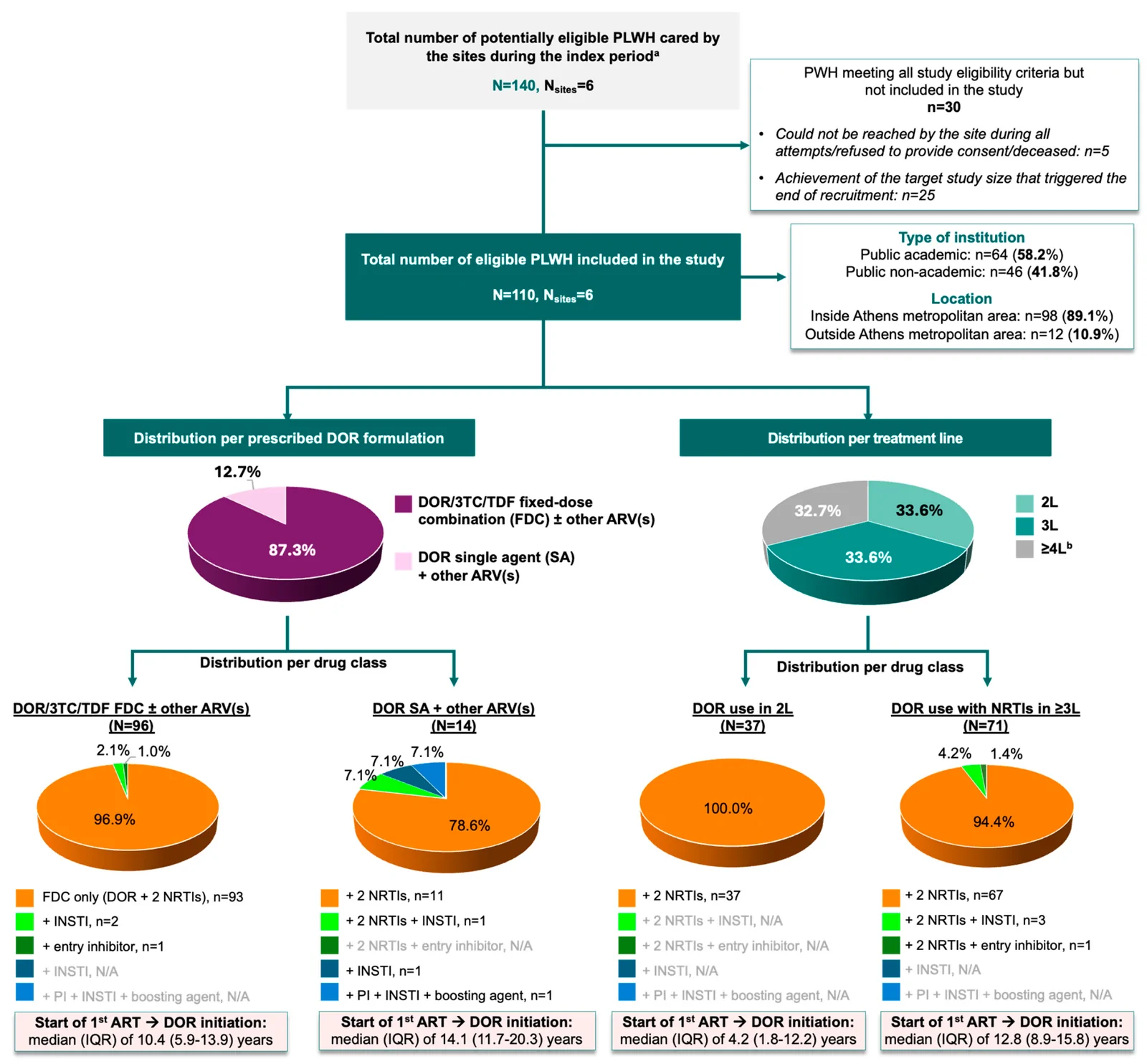
Disposition of participants and treatment characteristics. ^a^ Switched from their prior ART to a DOR-based regimen during the period between 04 February 2022 and the date corresponding to 30 days prior to site activation for study participation. ^b^ For the 2 PLWH who received DOR combined with ARV other than NRTIs in ≥3L, the median (IQR) time from start of 1st ART to DOR initiation was 10.7 and 27.5 years, respectively. Abbreviations: 2L/3L/7L, Second/Third/Seventh line; 3TC, Lamivudine; ART, Antiretroviral Therapy; ARV, Antiretroviral; DOR, Doravirine; FDC, Fixed-Dose Combination; INSTI, Integrase Strand Transfer Inhibitor; IQR, Interquartile Range; *n*, number of PLWH with variable; *N*, number of PLWH with available data in the medical records; N/A, not applicable; NRTI, Nucleos(t)ide Reverse Transcriptase Inhibitor; PI, Protease Inhibitor; PLWH, People with HIV; TDF, Tenofovir Disoproxil Fumarate; SA, Single Agent.

Over a total 3.6-month accrual period from 12 July 2023 to 31 October 2023, a total of 110 eligible PLWH were consecutively included by 6 public-sector hospital clinics (4 academic and 2 non-academic; 5 inside and 1 outside the Athens metropolitan area). Distribution of PLWH was balanced between non-academic and academic institutions, with 58.2% (64/110) and 41.8% (46/110) included by academic and non-academic clinics, respectively, whereas most PLWH (89.1%; 98/110) were included by sites located inside the Athens metropolitan area (Figure 1).

DOR was prescribed as a fixed-dose combination (FDC) with the NRTIs 3TC and TDF in the majority of PLWH (87.3%; 96/110) and as a single agent (SA) with other ARVs (‘DOR SA + other ARVs’) in the remaining 12.7% (14/110) (Figure 1). PLWH started DOR at a median (IQR) of 11.7 (6.6–14.3) years after first-ever ART initiation, with a median (IQR) number of prior ART regimens of 2.0 (1.0–3.0) and 66.4% (73/110) being prescribed in the third line (3L) or beyond (Figure 1). All but two PLWH (98.2%; 108/110) were prescribed DOR in combination with dual NRTI (with or without other ARV), of whom 34.3% (37/108) were in the 2L and 65.7% (71/108) in the ≥3L (Figure 1). In the two remaining PWLH, DOR was combined with ARVs other than NRTIs in ≥3L: one received DOR with an INSTI in the 4L, and the other received DOR combined with a protease inhibitor (PI), INSTI and boosting agent in the 7L setting. The proportion of PLWH who had received ≥3 prior ART regimens was 27.1% (26/96) among those treated with DOR/3TC/TDF fixed-dose combination and 71.4% (10/14) among those treated with SA + other ARV(s).

### 3.2. Profile of PLWH Switching to DOR-Based Regimen

Detailed sociodemographic, anthropometric and lifestyle characteristics of PLWH at baseline, overall, per prescribed DOR formulation and per ART line are presented in Table 1. Most PLWH were of white ethnicity (92.7%; 102/110), male sex at birth (90.9%; 100/110) and aged <65 years (91.8%; 101/110), with mean age being 43.1 and 52.0 years in those treated with ‘DOR + 2NRTIs in 2L’ (*N* = 37) and with ‘DOR + 2NRTIs (*N* = 71) in ≥3L’, respectively (Table 1).

At baseline, the median time since diagnosis was 13.0 years (6.0 years among those treated with ‘DOR + 2NRTIs in 2L’ and 15.3 years among those treated with ‘DOR + 2NRTIs in ≥3L’) (Table 2). The HIV clinical stage was asymptomatic for most PLWH (88.2%; 97/110), and among those evaluable, 86.5% (77/89) were virologically suppressed (defined as HIV-load < 50 copies/mL for at least 6 months prior to the date of first prescription of the DOR-based regimen).

Of the 110 participants, 86.4% (*n* = 95) had available immunological testing within 6 months before DOR initiation. The mean CD4+ cell absolute count was 697.2 cells/mm^3^ (Table 2), with 12.6% (12/95) and 3.2% (3/95) having ≤400 and <200 cells/mm^3^, respectively.

At baseline, the majority of the overall study population had at least one past/active clinically significant condition [median (IQR) Charlson comorbidity index (CCI) score: 1.0 (0.0–2.0)], with most being in the subgroup treated in ≥3L (94.5%; 69/73) (Table 3). Multimorbidity (≥2 clinically significant medical conditions) was also particularly high in this subgroup (42.5%; 31/73); a third of whom (32.9%; 24/73) were receiving lipid-lowering drugs and 15.1% (11/73) antihypertensives/heart failure (HF) agents. The respective proportions were numerically lower in the subgroup of ‘DOR + 2NRTIs in 2L’ [multimorbidity: 16.2% (6/37); lipid-lowering drugs: 8.1% (3/37), antihypertensives/HF agents: 2.7% (1/37)] (Table 3).

In the overall study population, HIV-1 genotypic testing within 12 months prior to the switch was performed in only seven PLWH (6.4%; 7/110); the relevant information on the HIV-1 genotyped regions and the mutations identified is presented in Table 4. Given the limited number of participants with available GRT results, these findings are descriptive only, and no inferences can be drawn regarding the prevalence of RAMs in the overall study cohort.

### 3.3. Reasons for Switching from Prior ART to a DOR-Based Regimen

**Figure 2:**
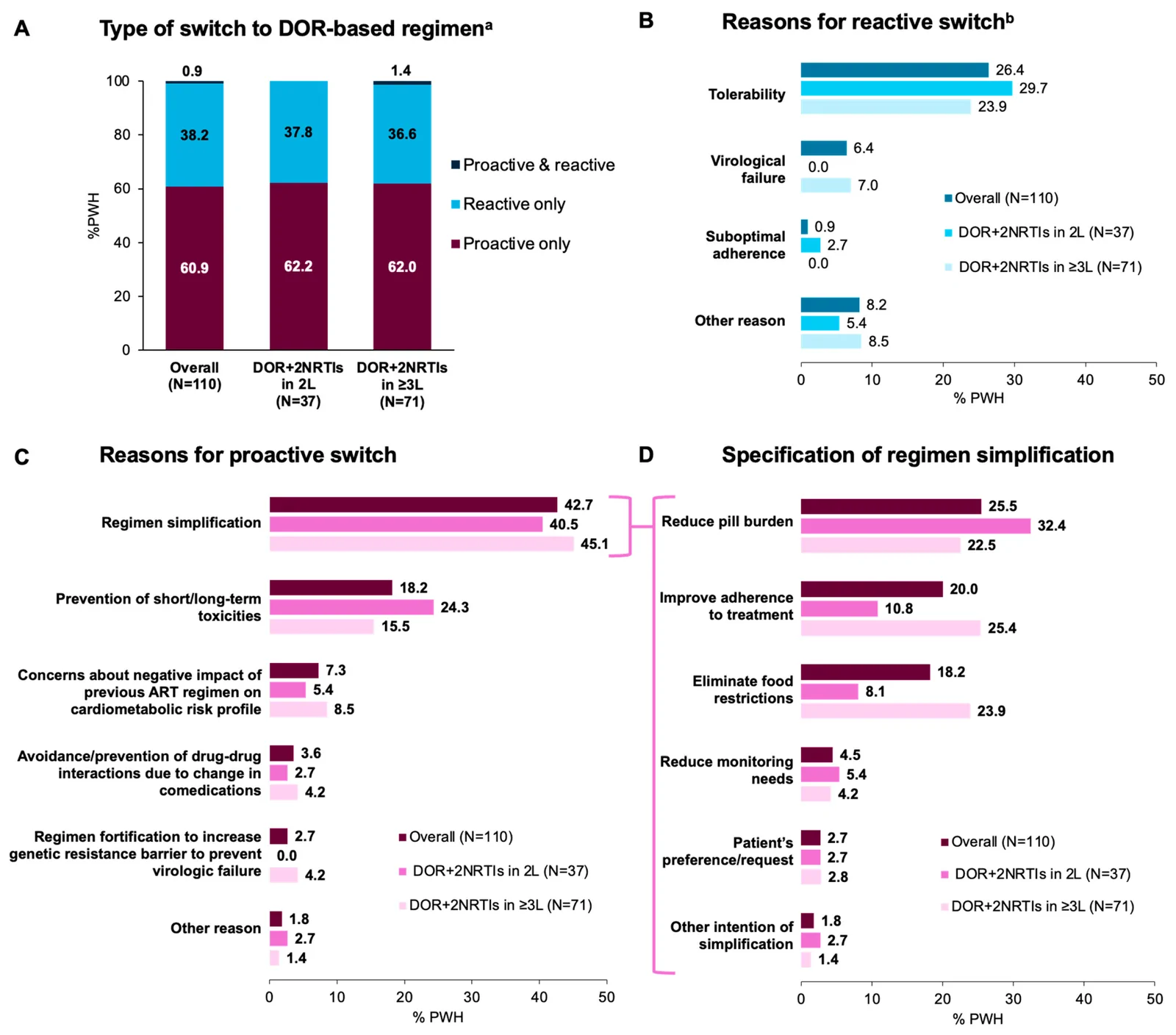
(**A**) Types and (**B**–**D**) reasons for switching from a prior ART to a DOR-based regimen. Reasons for switch to DOR-based regimen are not mutually exclusive; some PLWH have reported more than one reason for switch. ^a^ Both PLWH who received ‘DOR + other ARV in ≥3L’ were switched reactively. ^b^ The 2 PLWH who received ‘DOR + other ARV in ≥3L’ were switched reactively due to ‘virological treatment failure’ & ‘tolerability’ in one case, and ‘virological treatment failure’ & ‘other reason’ in the other case. Abbreviations: 2L/3L, Second/Third line; ART, Antiretroviral Therapy; ARV, Antiretroviral; DOR, Doravirine; *N*, number of PLWH with available data in the medical records; NRTI, Nucleos(t)ide Reverse Transcriptase Inhibitor; PLWH, People with HIV.

Approximately two-thirds of PLWH in the overall study population were proactively switched to a DOR-based regimen (61.8%; 68/110; 95% CI: 52.1–70.9%). This trend appeared similar between the subgroups of ‘DOR + 2NRTIs in 2L’ (62.2%; 23/37; 95% CI: 44.8–77.5%) and ‘DOR + 2NRTIs in the ≥3L’ (63.4%; 45/71; 95% CI: 51.1–74.5%) (Figure 2A). Reactive switching was reported in 39.1% (43/110; 95% CI: 29.9–48.9%) of the overall population, 37.8% (14/37; 95% CI: 22.5–55.2%) of ‘DOR + 2NRTIs in 2L’ and 38.0% (27/71; 95% CI: 26.8–50.3%) of ‘DOR + 2NRTIs in the ≥3L.’ For one patient in the latter subgroup, both proactive and reactive switches were recorded (1.4%; 1/71; 95% CI: 0.0–7.6%). Both participants in the ‘DOR + other ARV in ≥3L’ subgroup were switched reactively.

The most frequent reason for reactively switching to DOR was ‘tolerability’ in 26.4% (29/110), while a small proportion of PLWH (6.4%; 7/110) were switched due to ‘virological failure’ with the previous regimen, all of whom received ‘DOR in ≥3L’ (Figure 2B).

The most frequent reason for proactively switching to a DOR-based regimen was ‘regimen simplification’ in 42.7% (47/110) (Figure 2C). In the subgroup of ‘DOR + 2NRTIs in 2L’, the most frequent specification for ‘regimen simplification’ was to ‘reduce pill burden’ in almost a third (32.4%; 12/37). In the subgroup of ‘DOR + 2NRTIs in ≥3L’, the most frequent specifications for ‘regimen simplification’ were to ‘improve adherence to treatment’ and ‘eliminate food restrictions’ in 25.4% (18/71) and 23.9% (17/71), respectively (Figure 2D). Notably, for almost a fourth of PLWH who received ‘DOR + 2NRTIs in 2L’ (24.3%; 9/37), the reason for proactive switch was ‘prevention of short- or long-term toxicities’, whereas this proportion was numerically lower in the subgroup of ‘DOR + 2NRTIs in ≥3L’ (15.5%; 11/71) (Figure 2C). The two participants in the ‘DOR + other ARV in ≥3L’ subgroup were switched reactively due to ‘virological treatment failure’ and ‘tolerability’ in one case, and ‘virological treatment failure’ and ‘other reason’ in the other case.

## 4. Discussion

The DORAVITO study provides insight into real-life clinical decisions based on a well-characterized dataset of 110 treatment-experienced PLWH who switched to a DOR-based regimen in Greece. These findings contribute to the growing body of real-world evidence on DOR use in treatment-experienced PLWH, particularly in the Greek setting. They help address the existing gaps in the currently limited real-world data on the utilization of DOR-based treatment strategies and the characteristics of PLWH transitioning to this therapeutic option. Gaining insight into the factors influencing clinicians’ decisions to initiate a DOR-based regimen and identifying the profiles of appropriate candidates is essential for shaping evidence-based treatment strategies worldwide.

The study enrolled PLWH in 2023, at which point ample time had passed since local market access of DOR (February, 2022). Our findings thus reflect standard treatment practices by bypassing the early post-launch period during which selective prescription might create bias. Furthermore, six major national referral HIV centers, representing both academic and non-academic public institutions, participated in the study. As such, the study population was fairly representative and accounted for possible variations in medical practice paradigms.

By capturing data in the course of routine clinical care in Greece, we provide novel RWE on the demographic and clinical characteristics of PLWH switching to a DOR-based regimen as a therapeutic option as well as on the key determinants of treatment switching. In our study, DOR was prescribed in the ≥3rd line setting in 66% of PLWH and was co-administered with dual NRTI in all but two PLWH (98%), with the most commonly prescribed formulation being the FDC, in almost 9 out of 10 cases. Our cohort was mostly male, of white ethnicity, younger than 65 years, with low rates of AIDS or AIDS-defining conditions/illnesses and low rates of Hepatitis B and/or C virus. These characteristics are largely similar to the phase 3 DRIVE-SHIFT trial population, while our cohort was slightly older (median age of 49 versus 43 years in DRIVE-SHIFT), as would be expected based on the narrower trial eligibility criteria with respect to ART treatment line [28]. Furthermore, as opposed to the HIV-1 viral load restriction for entry into DRIVE-SHIFT, a non-negligible 14% of evaluable PLWH in DORAVITO were not virologically suppressed at baseline. This proportion is slightly higher than the general ART-treated HIV-infected population and other ART-experienced populations initiating DOR in the real world (7–9%) [8,35,36].

Metabolic issues and CV disorders constitute an increasingly important concern since PLWH live longer, leading to higher rates of multimorbidity and polypharmacy [5,6,7,46], which can be a challenge for clinicians aiming at reducing pill burden and DDIs. Similar to the general population in Greece, more than a third of our sample (38%) were active smokers (38% in the EMENO National Health Examination study) [47], while 46% reported moderate-vigorous physical activity (range of 32–52% in other Greek studies of the general population) [48,49]. However, continuing the exploratory examination of baseline characteristics, other metabolic and CV disease risk factors were less frequent in the DORAVITO cohort compared with the EMENO study population. In particular, obesity was reported in 11% versus 32%, diabetes mellitus in <5% versus 12%, dyslipidemia in 31% versus 60%, and hypertension in 13% versus 39%, even though median age was similar between the two studies (49 versus 48 years) [47]. Notably, a third (34%) of individuals had multimorbidity (excluding infections/infestations) and 6% were taking five or more comedications (i.e., polypharmacy). In Greek retrospective cohort and cross-sectional studies of PLWH (mean age range: 42–45 years; range of ART-treated: 84–99%; range of AIDS-defining illness: 11–14%; range of chronic viral hepatitis: 6–13%) [50,51,52], the rates of obesity (range: 11–14%) and diabetes mellitus (range: 5–7%) were similar to those observed in the DORAVITO cohort, whereas the rates of hypertension (range: 17–36%), dyslipidemia (range: 31–78%) and active smoking (range: 52–63%) were higher.

In line with the above observations, only 7% of PLWH were switched to DOR-based regimens in our cohort due to ‘concerns about negative impact of previous ART regimen on cardiometabolic risk profile.’ In fact, the higher proportion of proactive switchers compared with reactive switchers (60%:40%) was mainly driven by reasons of ‘regimen simplification’ (43%) and ‘prevention of toxicities’ (18%). A quarter of PLWH in DORAVITO (26%) switched to DOR from prior ART as a result of ‘tolerability’ issues, which was the most prevalent reason recorded for reactive switching.

Real-world studies conducted in European countries (Italy [25,35,41,42], England [36], Spain [37], and The Netherlands [38]) have affirmed the anticipated reasons for switching to a DOR-based regimen in treatment-experienced PLWH, including, among others, high CV/metabolic risk, dyslipidemia, weight gain, toxicity, DDIs, treatment simplification, and cost optimization/reduction. The reporting style across studies is highly variable. Nevertheless, to provide a context in which to interpret our findings, we touch on some similarities and differences between our data and other studies of similar or larger sample sizes [35,36,37,38] (excluding elderly people living with HIV [41,42]).

VF and DDI/pharmacological interaction as reasons for switch were relatively infrequent (6% and 4% in DORAVITO, respectively; 4–17% [35,37] and 5–13% [35,37,38] in other studies, respectively). On the other hand, the rate of ‘toxicity’/‘adverse event’ as reason for switch was 7% (Italy) [35], 14% (Spain) [37], and 38% (Netherlands) [38] across studies, with the latter also reporting 10% as ‘to prevent toxicity’. Compared with the latter Dutch study, which examines similar categories to DORAVITO, the reactive type of switch of ‘tolerability’ was less common (26%), while the proactive type of switch of ‘prevention of short/long-term toxicities’ was more frequent (18%).

Simplification (31%) was one of the main reasons for switch in the Dutch study [38] and the present Greek DORAVITO study (43%). Simplification, however, represented 17% of switchers in the English DRIVE-REAL study [36] and was not mentioned in the other 2 studies [35,37]. ‘Prevention of metabolic complications’ and ‘dyslipidemia’ were reported in a high proportion of the Italian study cohort (39% and 18%, respectively) [35]. This trend was not observed in other studies (not mentioned) [36,37,38] nor the Greek DORAVITO study (7% were concerned about cardiometabolic risk). However, the Italian cohort was older (median of 56 years) and had been diagnosed with HIV for a longer time (median of 22 years) [35,41] than DORAVITO. Lastly, economic/financial reasons were reported in a high proportion of the Spanish study (44%) [37], and 10% of the Dutch cohort. The respective proportion in DORAVITO was <2% (by extrapolation of the ‘Other proactive’ category) and 0% in the English DRIVE-REAL study [36].

Overall, the rationale for switching to DOR in Greece seems largely aligned with the Dutch nationwide cohort [38]. This observation is based on our naive cross-country comparison lacking any statistical support, thus should be cautiously interpreted. Conclusions cannot be inferred with respect to the English DRIVE-REAL multicenter cohort due to the limited categorization available [36]. While differences compared with the Italian [35] and Spanish cohort [37] may be partly attributed to study design (e.g., they were both single-center studies, thus may lack generalizability). Notwithstanding, characteristics of PLWH may vary between countries and, as a result, the rationale for changing an otherwise effective ART regimen may be influenced. Therefore, local real-world data such as those presented herein are necessary to better inform healthcare policies and support informed clinical decisions.

The main limitations of this study are attributed to its retrospective chart review design and mainly involve systematic errors. Sampling bias is considered minimal given the consecutive enrolment process that was implemented and the fact that only five potentially eligible PLWH were not included. Furthermore, the overall included eligible population corresponded to 79% (110/140) of the total number of PLWH cared for by these sites who switched from their prior ART to a DOR-based regimen during the site-specific index period (Figure 1). Regarding baseline characteristics, the lack of data rate did not exceed 10% except for illicit/recreational drug use (16%), virologic suppression status (19%) and CD4+ cell count (14%). However, interpretation of virological and immunological characteristics should take into consideration the possibility of interlaboratory bias which is inherent to routine clinical settings.

Another limitation of this study stems from the lack of data on the specific ARVs used immediately before the switch. However, this constraint is inherent to the study design, as prior ARV regimens were not collected because they were beyond the study’s scope. In addition, in the absence of a comparator group, the study was not designed to address whether DOR is superior, equivalent, or inferior to alternative regimens in real-world practice. These, along with possible recording bias, mean that the reasons for switching may not capture the full complexity of clinical decision-making.

Furthermore, as this was a retrospective study, only GRT results documented in the medical records could be captured. Consequently, results within 12 months before treatment switch were available for only seven study participants. While the possibility of documentation bias cannot be excluded, the limited availability of GRT may reflect the clinical context of switching to DOR-based regimens, in which GRT is not routinely undertaken close to treatment switch. This is consistent with virological failure not being a common reason for treatment switch in this cohort. Therefore, this exploratory analysis should be considered descriptive and primarily hypothesis-generating rather than representative of the resistance profile of the overall study cohort.

Lastly, the overall study population size of 110 PLWH met the originally planned size. Nevertheless, the study subpopulations that were formed to address the secondary study objectives comprised less than 100 individuals, with the resulting subgroups being extremely small in some cases; in particular, the subgroup switching to DOR combined with ARVs other than NRTI (*N* = 2), and the subgroup prescribed SA DOR (‘DOR SA + other ARVs’; *N* = 14). In these cases, the precision of estimates and meaningfulness of derived inferences may be compromised. Formal comparisons between subgroups were out of scope of this study; thus, any differences are mentioned in a purely descriptive manner and should be interpreted with caution.

## 5. Conclusions

This study describes the baseline characteristics and reported reasons for treatment switching among Greek PLWH initiated on DOR-based regimens in routine public-sector clinical practice. The most common reasons for switching were ‘regimen simplification’, ‘tolerability’ and ‘prevention of toxicities’. In view of the paucity of real-world data regarding the DOR prescription landscape in Greece, our findings will support future clinical decision-making.

## Figures and Tables

**Table 1:** Sociodemographic, anthropometric and lifestyle characteristics of PLWH at baseline.

	Overall (*N* = 110)	Prescribed DOR Formulation		ART Treatment Line		
		FDC ± Other ARV (*N* = 96)	SA + Other ARV (*N* = 14)	DOR + 2 NRTIs in 2L (*N* = 37)	DOR + 2 NRTIs in ≥3L (*N* = 71)	DOR + Other ARV in ≥3L (*N* = 2)
Age, mean (SD), ^a^ years	49.3 (10.8)	48.6 (10.5)	54.6 (11.5)	43.1 (11.2)	52.0 (8.2)	53.0/88.0 ^b^
Age < 65 years, % (*n*/*N*)	91.8 (101/110)	91.7 (88/96)	92.9 (13/14)	94.6 (35/37)	91.5 (65/71)	50.0 (1/2)
Male sex assigned at birth, % (*n*/*N*)	90.9 (100/110)	91.7 (88/96)	85.7 (12/14)	86.5 (32/37)	93.0 (66/71)	100.0 (2/2)
Greek ethnic origin, % (*n*/*N*)	75.5 (83/110)	75.0 (72/96)	78.6 (11/14)	59.5 (22/37)	83.1 (59/71)	100.0 (2/2)
Employed, % (*n*/*N*)	72.4 (76/105)	72.0 (67/93)	75.0 (9/12)	75.7 (28/37)	71.2 (47/66)	50.0 (1/2)
Living alone, % (*n*/*N*)	66.7 (68/102)	65.9 (58/88)	71.4 (10/14)	54.1 (20/37)	73.0 (46/63)	100.0 (2/2)
Public health insurance coverage, % (*n*/*N*)	92.7 (102/110)	92.7 (89/96)	92.9 (13/14)	89.2 (33/37)	94.4 (67/71)	100.0 (2/2)
BMI, mean (SD), kg/m^2^	25.2 (3.8)	25.3 (3.8)	24.7 (4.1)	24.1 (3.6)	25.9 (3.8)	18.3/23.5 ^b^
Obese (BMI ≥ 30 kg/m^2^), % (*n*/*N*)	11.4 (12/105)	11.0 (10/91)	14.3 (2/14)	5.6 (2/36)	14.9 (10/67)	
Current/former smokers, ^c^ % (*n*/*N*)	61.8 (68/110)	61.5 (59/96)	64.3 (9/14)	54.1 (20/37)	66.2 (47/71)	50.0 (1/2)
Current/former illicit/recreational drug users, % (*n*/*N*)	22.6 (21/93)	24.7 (20/81)	8.3 (1/12)	20.6 (7/34)	24.6 (14/57)	
Moderate/vigorous physical activity, % (*n*/*N*)	45.8 (49/107)	47.8 (43/90)	42.9 (6/14)	56.8 (21/37)	39.7 (27/68)	50.0 (1/2)
Adequate dietary/nutrient intake level, % (*n*/*N*)	98.1 (106/108)	97.9 (93/95)	100.0 (13/13)	100.0 (36/36)	97.1 (68/70)	100.0 (2/2)

^a^ Median age was 49.0. ^b^ For these two PLWH the exact values are presented. ^c^ 38.2% (42/110) were active smokers at baseline. Abbreviations: 2L/3L, Second/Third line; ART, Antiretroviral Therapy; ARV, Antiretroviral (agent); BMI, Body Mass Index; DOR, Doravirine; FDC, fixed-dose combination; *n*, number of PLWH with variable; *N*, number of PLWH with available data in the medical records; NRTI, Nucleos(t)ide Reverse Transcriptase Inhibitor; PLWH, People with HIV; SA, Single Agent; SD, Standard Deviation.

**Table 2:** Clinical, virological, and immunological characteristics.

		Overall (*N* = 110)	Prescribed DOR Formulation		ART Treatment Line		
			FDC ± Other ARV (*N* = 96)	SA + Other ARV (*N* = 14)	DOR + 2 NRTIs in 2L (*N* = 37)	DOR + 2 NRTIs in ≥3L (*N* = 71)	DOR + Other ARV in ≥3L (*N* = 2)
**At initial HIV-1 diagnosis**							
Age at diagnosis, median (IQR), years		35.0 (29.0–41.0)	35.0 (29.0–41.5)	34.5 (29.0–41.0)	33.0 (28.0–42.0)	35.0 (29.0–41.0)	26.0/60.0 ^a^
Sexual transmission of HIV-1, % (*n*/*N*)		93.5 (101/108)	93.6 (88/94)	92.9 (13/14)	94.3 (33/35)	93.0 (66/71)	100.0 (2/2)
**At baseline**							
Duration of HIV-1 disease, median (IQR), years		13.0 (7.2–16.8)	12.3 (6.4–16.3)	16.4 (13.3–22.7)	6.0 (2.5–13.1)	15.3 (11.0–20.4)	27.5/28.6 ^a^
HIV clinical stage, % (*n*/*N*)	Asymptomatic	88.2 (97/110)	87.5 (84/96)	92.9 (13/14)	91.9 (34/37)	85.9 (61/71)	100.0 (2/2)
	Any AIDS-defining condition	8.2 (8/97)	7.1 (6/84)	15.4 (2/13)	5.9 (2/34)	9.8 (6/61)	
	Symptomatic (without AIDS-defining conditions)	8.2 (9/110)	8.3 (8/96)	7.1 (1/14)	5.4 (2/37)	9.9 (7/71)	
	AIDS	3.6 (4/110)	4.2 (4/96)		2.7 (1/37)	4.2 (3/71)	
Viral load above the LLOQ in the last 6 months, % (*n*/*N*)		17.4 (16/92)	16.7 (13/78)	21.4 (3/14)	13.9 (5/36)	16.7 (9/54)	100.0 (2/2)
Virologically suppressed, ^b^ % (*n*/*N*)		86.5 (77/89)	86.7 (65/75)	85.7 (12/14)	84.8 (28/33)	90.7 (49/54)	
CD4+ absolute count (cells/mm^3^), ^c^ mean (SD)		697.2 (304.6)	689.1 (307.4)	744.2 (294.2)	628.6 (275.7)	738.7 (317.5)	440.6/943.3 ^a^

^a^ For these two PLWH the exact values are presented; ^b^ Defined as HIV-load < 50 copies/mL for at least 6 months prior to first DOR prescription; ^c^ Among those with available immunological testing in the last 6 months. Abbreviations: 2L/3L, Second/Third line; ART, Antiretroviral Therapy; ARV, Antiretroviral (agent); DOR, Doravirine; FDC, fixed-dose combination; IQR, Interquartile Range; LLOQ, Lower Limit Of Quantification; *n*, number of PLWH with variable; *N*, number of PLWH with available data in the medical records; NRTI, Nucleos(t)ide Reverse Transcriptase Inhibitor; PLWH, People with HIV; SA, Single Agent; SD, Standard Deviation.

**Table 3:** Comorbid conditions and non-HIV comedications at baseline.

		Overall (*N* = 110)	Prescribed DOR Formulation		ART Treatment Line		
			FDC ± Other ARV (*N* = 96)	SA + Other ARV (*N* = 14)	DOR + 2 NRTIs in 2L (*N* = 37)	DOR + 2 NRTIs in ≥3L (*N* = 71)	DOR + Other ARV in ≥3L (*N* = 2)
**Clinically significant medical/surgical history/comorbid conditions including history of AIDS-defining illnesses, opportunistic infections and other co-infections at baseline**							
≥1 disease/condition/surgery, % (*n*/*N*)		87.3 (96/110)	87.5 (84/96)	85.7 (12/14)	73.0 (27/37)	94.4 (67/71)	100.0 (2/2)
≥2 diseases/conditions/surgeries, % (*n*/*N*)		59.1 (65/110)	56.3 (54/96)	78.6 (11/14)	40.5 (15/37)	67.6 (48/71)	100.0 (2/2)
≥3 diseases/conditions/surgeries, % (*n*/*N*)		28.2 (31/110)	26.0 (25/96)	42.9 (6/14)	13.5 (5/37)	35.2 (25/71)	50.0 (1/2)
Most frequent conditions (frequency ≥ 5.0% overall), % (*n*/*N*)	Dyslipidaemia	30.9 (34/110)	28.1 (27/96)	50.0 (7/14)	16.2 (6/37)	38.0 (27/71)	50.0 (1/2)
	Syphilis	27.3 (30/110)	26.0 (25/96)	35.7 (5/14)	21.6 (8/37)	31.0 (22/71)	
	Hypertension	12.7 (14/110)	13.5 (13/96)	7.1 (1/14)	2.7 (1/37)	18.3 (13/71)	
	Papilloma viral infection	9.1 (10/110)	9.4 (9/96)	7.1 (1/14)	10.8 (4/37)	8.5 (6/71)	
	Anxiety	7.3 (8/110)	7.3 (7/96)	7.1 (1/14)	5.4 (2/37)	8.5 (6/71)	
	Hepatitis C	5.5 (6/110)	5.2 (5/96)	7.1 (1/14)	8.1 (3/37)	4.2 (3/71)	
	Lymphadenopathy	5.5 (6/110)	5.2 (5/96)	7.1 (1/14)	5.4 (2/37)	4.2 (3/71)	50.0 (1/2)
HCV, % (*n*/*N*)		5.5 (6/110)	5.2 (5/96)	7.1 (1/14)	8.1 (3/37)	4.2 (3/71)	
HBV, % (*n*/*N*)		3.6 (4/110)	3.1 (3/96)	7.1 (1/14)		5.6 (4/71)	
Other opportunistic infections/co-infections, % (*n*/*N*)		47.3 (52/110)	46.9 (45/96)	50.0 (7/14)	35.1 (13/37)	54.9 (39/71)	
Multimorbidity (≥2 clinically significant medical conditions; excluding infections/infestations), % (*n*/*N*)		33.6 (37/110)	30.2 (29/96)	57.1 (8/14)	16.2 (6/37)	40.8 (29/71)	100.0 (2/2)
CCI score ≥ 3 (moderate/high comorbidity burden), % (*n*/*N*)		15.5 (17/110)	14.6 (14/96)	21.4 (3/14)	8.1 (3/37)	18.3 (13/71)	50.0 (1/2)
Cardiometabolic CCI score, ^a^ median (IQR)		0.0 (0.0–1.0)	0.0 (0.0–1.0)	1.0 (0.0–2.0)	0.0 (0.0–0.0)	1.0 (0.0–1.0)	1.0/6.0 ^c^
Non-cardiometabolic CCI score, ^b^ median (IQR)		1.0 (0.0–2.0)	1.0 (0.0–1.5)	1.0 (1.0–2.0)	0.0 (0.0–1.0)	1.0 (0.0–2.0)	1.0/6.0 ^c^
**Non-HIV comedications at baseline**							
Treatment of comorbidities or for unknown reasons, % (*n*/*N*)		45.5 (50/110)	44.8 (43/96)	50.0 (7/14)	27.0 (10/37)	53.5 (38/71)	100.0 (2/2)
Most frequent (frequency ≥ 5.0% overall), % (*n*/*N*)	Lipid-lowering drugs	24.5 (27/110)	24.0 (23/96)	28.6 (4/14)	8.1 (3/37)	32.4 (23/71)	50.0 (1/2)
	Antihypertensives/HF agents		10.9 (12/110)	12.5 (12/96)		2.7 (1/37)	15.5 (11/71)
	Antidepressants/anxiolytics		7.3 (8/110)	7.3 (7/96)	7.1 (1/14)	8.1 (3/37)	7.0 (5/71)
	Antithrombotics		7.3 (8/110)	7.3 (7/96)	7.1 (1/14)		11.3 (8/71)
Prophylaxis of opportunistic infections (other than hepatitis infections), % (*n*/*N*)		5.5 (6/110)	5.2 (5/96)	7.1 (1/14)	5.4 (2/37)	4.2 (3/71)	50.0 (1/2)
Polypharmacy rate (≥5 non-HIV comedications; excluding non-conventional medication), % (*n*/*N*)		5.5 (6/110)	6.3 (6/96)		2.7 (1/37)	7.0 (5/71)	
Non-conventional medications for any reason, % (*n*/*N*)		11.0 (11/100)	11.4 (10/88)	8.3 (1/12)	13.9 (5/36)	9.7 (6/62)	

^a^ Including: myocardial infarction, congestive heart failure, peripheral vascular disease, cerebrovascular disease, diabetes mellitus, renal disease [Type 2 diabetes mellitus was reported in 3.6% (4/110)]; ^b^ Including: dementia, connective tissue disorder, peptic ulcer disease, cancer (hematological malignancies and solid tumors), hemiplegia, liver disease, chronic pulmonary disease; ^c^ For these two PLWH the exact values are presented. Abbreviations: 2L/3L, Second/Third line; ART, Antiretroviral Therapy; ARV, Antiretroviral (agent); CCI, Charlson Comorbidity Index; DOR, Doravirine; FDC, fixed-dose combination; HBV, Hepatitis B virus; HCV, Hepatitis C Virus; HF, Heart Failure; *n*, number of PLWH with variable; *N*, number of PLWH with available data in the medical records; NRTI, Nucleos(t)ide Reverse Transcriptase Inhibitor; SA, Single Agent.

**Table 4:** HIV-1 resistance-associated mutations, overall, per pol gene segment (RT, PR and IN) of the HIV-1 genome and per clinical significance, in the subgroup tested within 12 months prior to switch to DOR-based regimen.

		% (*n*/*N*)
**HIV-1 genotypic resistance testing within 12 months prior to switch to DOR-based regimen**		**6.4 (7/110)**
Regions of HIV-1 genotyped	RT, PR and IN	57.1 (4/7)
	RT and PR	42.9 (3/7)
**Mutations identified among PLWH who had undergone genotyping of the RT region (*N* = 7)**		
Any mutation identified		57.1 (4/7)
Presence of any **mutation of major clinical significance (indicated in bold)**		28.6 (2/7)
NNRTI resistance mutation		57.1 (4/7)
V179I		28.6 (2/7)
E138G, K103R, V106I, V179D		14.3 (1/7), each
NRTI resistance mutation		28.6 (2/7)
**M184V**		28.6 (2/7)
**D67N, L74V, Y115F,** K70N, K70Q		14.3 (1/7), each
**Mutations identified among PLWH who had undergone genotyping of the PR region (*N* = 7)**		
Any mutation identified		71.4 (5/7)
Non-Major PI mutation		71.4 (5/7)
K20R, M36I		42.9 (3/7), each
I62V, I64M, I93L, L89M, V77I		28.6 (2/7), each
A71T, D60E, H69K, I64V, L10F, L63P, V82I		14.3 (1/7), each
**Mutations identified among PLWH who had undergone genotyping of the IN region (*N* = 4)**		
Any mutation identified		50.0 (2/4)
**Major INSTI mutation**		50.0 (2/4)
**E138K, S147G**		50.0 (2/4), each
**Q148R, T66A**		25.0 (1/4), each
Non-Major INSTI mutation		50.0 (2/4)
E157Q, L74I, T97A, V72I		25.0 (1/4), each

Abbreviations: DOR, doravirine; IN, Integrase; INSTI, Integrase Strand Transfer Inhibitor; *n*, number of PLWH with variable; *N*, number of PLWH with available data in the medical records; NNRTI, Non-Nucleoside Reverse Transcriptase Inhibitor; NRTI, Nucleos(t)ide Reverse Transcriptase Inhibitor; PI, Protease Inhibitor; PLWH, People Living with HIV; PR, Protease; RT, Reverse Transcriptase.

## Data Availability

The data that support the findings of this study are not publicly available due to their containing information that could compromise the privacy of PLWH included in the study. For any request related to data and materials, please contact the corresponding author.
